# Pathobiological Characterization of a Novel Reassortant Highly Pathogenic H5N1 Virus Isolated in British Columbia, Canada, 2015

**DOI:** 10.1038/srep23380

**Published:** 2016-03-18

**Authors:** Yohannes Berhane, Darwyn Kobasa, Carissa Embury-Hyatt, Brad Pickering, Shawn Babiuk, Tomy Joseph, Victoria Bowes, Mathew Suderman, Anders Leung, Colleen Cottam-Birt, Tamiko Hisanaga, John Pasick

**Affiliations:** 1 Canadian Food Inspection Agency, National Centre for Foreign Animal Disease, Winnipeg, Manitoba, Canada R3E 3M4; 2 Department of Animal Science, University of Manitoba, Winnipeg, Canada; 3 Public Health Agency of Canada, National Microbiology Laboratory, 1015 Arlington Street, Winnipeg, Manitoba, Canada; 4 Department of Medical Microbiology, University of Manitoba, Winnipeg, Canada; 5 Department of Immunology, University of Manitoba, Winnipeg, Canada; 6 Animal Health Centre, Ministry of Agriculture, Abbotsford, British Columbia, Canada V3G 2M3

## Abstract

In the current study, we describe the pathobiologic characteristics of a novel reassortant virus - A/chicken/BC/FAV-002/2015 (H5N1) belonging to clade 2.3.4.4 that was isolated from backyard chickens in British Columbia, Canada. Sequence analyses demonstrate PB1, PA, NA and NS gene segments were of North American lineage while PB2, HA, NP and M were derived from a Eurasian lineage H5N8 virus. This novel virus had a 19 amino acid deletion in the neuraminidase stalk. We evaluated the pathogenic potential of this isolate in various animal models. The virus was highly pathogenic to mice with a LD_50_ of 10 plaque forming units (PFU), but had limited tissue tropism. It caused only subclinical infection in pigs which did result in seroconversion. This virus was highly pathogenic to chickens, turkeys, juvenile Muscovy ducks (*Cairnia moschata foma domestica*) and adult Chinese geese (*Anser cynoides domesticus*) causing a systemic infection in all species. The virus was also efficiently transmitted and resulted in mortality in naïve contact ducks, geese and chickens. Our findings indicate that this novel H5N1 virus has a wide host range and enhanced surveillance of migratory waterfowl may be necessary in order to determine its potential to establish itself in the wild bird reservoir.

In December 2014, a highly pathogenic avian influenza (HPAI) outbreak due to a novel reassortant H5N2 virus was reported near Abbotsford, British Columbia^1^. This outbreak was extraordinary in that it was the first reported case involving the transcontinental spread of Eurasian A/Goose/Guangdong/1/96- like HPAI H5N1 lineage (Gs/Gd like-lineage) viruses to North America. Eurasian Gs/Gd like-lineage HPAI H5N1 first emerged in domestic geese in south-eastern China in 1996 eventually spreading to infect domestic poultry and wild birds in Asia, Europe and Africa^2^,^3^,^4^,^5^. Currently viruses of the Gs/Gd like-lineage have become endemic in poultry in a number of countries in Asia and Africa. Since their emergence, Gs/Gd like-lineage H5N1 viruses have caused huge economic losses to the poultry industry as a result of mortalities and stamping out activities^3^,^6^,^7^. In addition, these viruses as of 15 November, 2015, have been associated with 844 human cases and an associated case fatality rate of ~53% resulting primarily from direct contact with infected birds (http://www.who.int/influenza/human_animal_interface/H5N1_cumulative_table_archives/en/). Since 2003 the hemagglutinin (HA) gene of Gs/Gd like-lineage H5N1 viruses has undergone continual evolutionary divergence resulting in the establishment of distinct phylogenetic clades^8^. This prompted the WHO/OIE/FAO H5N1 Evolution Working Group in 2008^9^ to establish a system of classifying these divergent groups.

Avian influenza (AI) virus surveys and surveillance have been conducted in North American wild waterfowl populations for a number of years; one of the objectives being to assess whether Gs/Gd-like HPAI H5N1 viruses could be introduced to the Americas via intermingling of long range migratory birds along East Asian-Australasian, West Pacific and Pacific Americas migratory flyways. Although there was no evidence for Gs/Gd-like HPAI H5N1 introduction, some studies did show evidence of limited intermingling of Eurasian and American AI viruses along these migratory flyways^10^,^11^,^12^,^13^,^14^,^15^.

This changed with the recent emergence of Gs/Gd-like H5N8 viruses belonging to clade 2.3.4.4. Early in 2014, outbreaks of HPAI H5N8 virus were reported in China, South Korea, Japan and Russia where migratory birds were suspected to have played a key role in the introduction of this virus to domestic poultry^16^. During the fall of 2014, this H5N8 virus spread to Western European countries causing outbreaks in Germany, the Netherlands, England and Italy^17^. In December 2014, an outbreak of HPAI caused by a novel reassortant H5N2 virus with a Gs/Gd-like HA gene belonging to clade 2.3.4.4 was reported in the lower Fraser Valley of British Columbia, Canada^1^. This virus consisted of a mixture of gene segments of Eurasian HPAI H5N8 virus and North American lineage low pathogenic virus origin. This HPAI H5N2 virus caused outbreaks in 12 different poultry premises in the lower Frazer Valley (data not shown). A wholly Eurasian H5N8 virus was subsequently detected in wild waterfowl in the same area. Wholly Eurasian H5N8 and reassortant H5N2 viruses were eventually detected in captive birds, wild birds and backyard domestic poultry flocks in Washington, Oregon and California (USDA, APHIS data base). This was followed by outbreaks of H5N2 and H5N8 in commercial domestic poultry, initially in states located along the Pacific flyway, with further outbreaks of H5N2 in states located along Central and Mississippi flyways^18^. In early April 2015, three HPAI H5N2 outbreaks in domestic poultry (2 turkey and 1 chicken farms) were reported in Oxford county, Ontario, Canada. All three of these farms were located on the extreme eastern edge of the Mississippi flyway.

On December 24, 2014, a second novel reassortant HPAI H5N1 virus was isolated from a green-winged teal (*Anas Crecca*) shot by a hunter in Whatcom County, Washington^19^. In February 2015, a similar HPAI H5N1 virus was isolated from backyard chicken layer flock in the lower Fraser Valley, British Columbia. Although animal studies have been conducted in a number of different species using Eurasian clade 2.3.4.4 H5N8 viruses^6^,^7^,^20^,^21^, little was known about the pathogenic potential of this novel HPAI H5N1 in different species of birds and mammals. While a closely related novel H5N1 virus, A/American green-winged teal/Washington/ 195750/2014 (AGT/H5N1), had been isolated from a hunter killed green-winged teal^19^, no information was available regarding the clinical and pathological presentation of the affected animal. Previous reports involving natural or experimental infections with Gs/Gd-like HPAI H5N1 viruses showed that they could replicate and efficiently transmit in ducks, however, the pathogenicity ranged widely from sub-clinical infection to clinical disease with mortality.

Here we assess the pathobiological characteristics of a novel reassortant HPAI H5N1 virus that was isolated from a backyard chicken flock in domestic gallinaceous poultry, domestic geese, domestic ducks, pigs and mice. The aim of this study was to help evaluate the risk associated with this new virus to birds and mammals while also gaining a clearer understanding of the infectivity, pathogenicity and clinical signs in different species.

## Results

### Genetic characterization of the novel HPAI H5N1 reassortant virus

The full genome sequence of the virus was acquired directly from lung tissue that was collected from clinically sick or dead chickens as well as from a virus isolate obtained after passaging in embryonated chicken eggs (FAV-002/H5N1). Alignment of full genome sequences obtained from the 3 tissue samples and the virus isolate were 99.8% to 100% identical to one another. The full genome sequence of FAV-002/H5N1 has been deposited in the NCBI data base with accession numbers KP892988 to KP892995. Sequence analyses demonstrated that 4 gene segments - basic polymerase 1 (PB1), acidic polymerase (PA), neuraminidase (NA) and non-structural (NS) were derived from an unknown virus of North American lineage; the basic polymerase 2 (PB2), hemagglutinin (HA), nucleoprotein (NP) and matrix (M) segments were derived from a Eurasian lineage H5N8 virus. The derived HA_0_ cleavage site NSPLRERRRKR/GLF was found to be consistent with other Gs/Gd like-lineage Eurasian H5 subtype viruses belonging to clade 2.3.4.4. Top NCBI BLAST matches for each gene segment of FAV-002/H5N1 are summarized in Table 1. When compared to AGT/H5N1, its closest match in the NCBI database, FAV-002/H5N1 had a number of non-synonymous mutations. These included: a V to A substitution involving the 14^th^ amino acid of the signal peptide of the HA precursor and E501D in the HA_2_; A21T in NA; L522M in PB2; E78D in PB1; and H271P and D347N in PA. No mutations were observed in the NP, M and NS gene segments.

**Figure 1:**
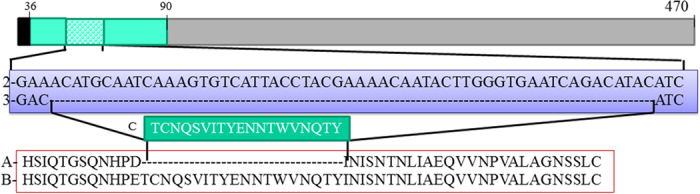
Schematic description of full neuraminidase type 1 protein – 470 amino acids (1). The NA domain corresponding to the stalk region is highlighted in green (36–90 AA) and the deletion of 19 AA in FAV-002 /H5N1 is highlighted in green/white squares. The globular head is highlighted in grey and the transmembrane domain in black. Alignment of the nucleotide sequence of the stalk region which is deleted in FAV-002/H5N1 (3) and AGT/H5N1 (2). Alignment of the protein sequence stalk region of FAV-002/H5N1 (A) and AGT/H5N1 (B). The deleted part of the NA stalk of FAV-002/H5N1 (C) is highlighted in green box.

The most notable change in FAV-002/H5N1 involved the NA protein which had a 19 AA deletion (48-TCNQSVITY ENNTWVNQTY-66) in its stalk when compared to AGT/H5N1 (Fig. 1). This 19 AA deletion in the NA of FAV-002/H5N1 compared to AGT/H5N1, led us to further investigate whether the infected backyard flock contained a mixed population of H5N1 viruses with deleted and non-deleted NA stalk regions. Following deep sequencing of the NA gene amplified from the 12 original lung tissue specimens collected from the backyard flock as well as the FAV-002/H5N1 isolate, no full length NA genes were found.

### Pathobiological properties of FAV-002/H5N1 in domestic poultry and waterfowl

#### Chickens

**Figure 2:**
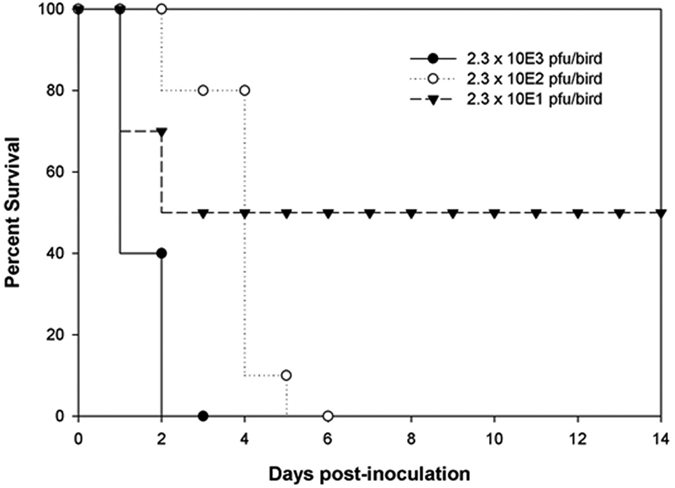
The survival curves for SPF chickens infected with three different dilutions of FAV-002/H5N1. The LD_50_ of FAV-002/H5N1 in chickens was determined to be 23 PFU.

The intravenous pathogenicity index (IVPI) of FAV-002/H5N1 was determined to be 2.97 indicating a HPAI virus. IVPI scores can range from 0 where none of the birds exhibit signs of disease during the 10 day observation period, to 3.00 where all of the birds die within the first 24 hours post-inoculation. The threshold score for considering a virus to be highly pathogenic is 1.2. The LD_50_ of FAV-002/H5N1 in chickens was determined to be 23 plaque forming units (PFU) (Fig. 2).

#### Turkeys

**Figure 3:**
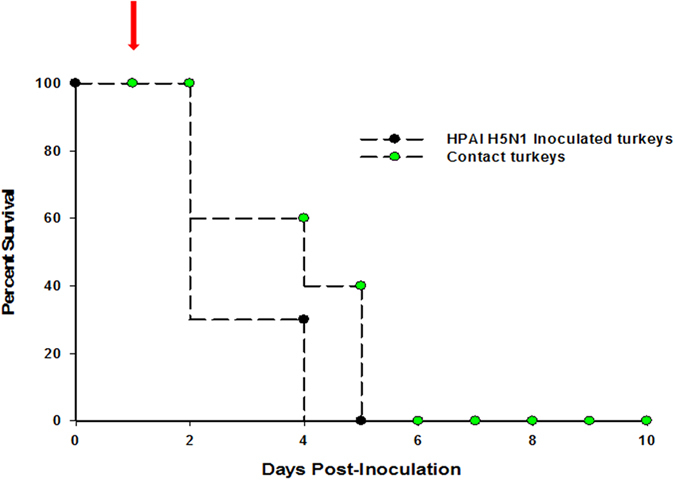
The survival curves for domestic juvenile turkeys infected with 1000 PFU of FAV-005/H5N1 and naïve contact turkeys that were placed among the infected 24 hrs after infection. The arrow indicates when naïve contact turkeys were placed among the infected turkeys at 24 hrs post-infection.

FAV-002/H5N1 was very lethal to juvenile domestic turkeys. Four turkeys died at 2 dpi following intranasal inoculation with 10^3^ PFU of virus and 1 contact turkey died 24 hrs post-contact without showing any clinical signs. The remaining turkeys were very sick and showed clinical signs that included ruffled feathers, drooped wings, depression, labored breathing, oral discharge, and neurological abnormalities such as ataxia and head shaking. Some of the feces (25%) contained blood. The remaining turkeys died or were euthanized due to severe illness by 4 dpi. The major postmortem findings included fibrinous pleuritis in lungs, swollen kidneys, mottled spleens with large white necrotic areas, large white focal necrotic areas in the pancreas, and an enlarged friable liver with white areas of necrosis or pinpoint areas of hemorrhage. The survival curve for the inoculated and contact turkeys is shown in Fig. 3.

#### Chinese Geese

**Figure 4:**
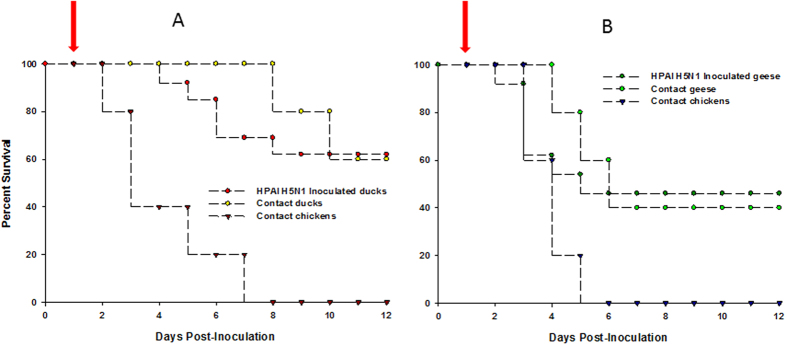
Survival curves of birds infected with 10,000 PFU of FAV-002/H5N1 and their contacts. (**A**) shows the survival curves of Muscovy ducks that were infected with 10,000 PFU of FAV-002/H5N1. The arrow indicates when Muscovy ducks and chickens that were placed among the infected ducks at 24 hrs post-infection. (**B**) shows the survival curves of Chinese geese that were infected with 10,000 PFU of FAV-002/H5N1 and contact Chinese geese and chickens that were placed in the same animal cubicle at 24 hrs post-infection (arrow).

FAV-002/H5N1 was moderately pathogenic to Chinese geese following inoculation by oronasal and cloacal routes. Clinical signs first appeared in the infected geese at 3 dpi and lasted until 9 dpi. The clinical signs included green feces, trembling, depression/lethargy, ruffled feathers, huddling, labored breathing, inability to walk when approached and drooping of the wings. Seven of the nine inoculated geese showed signs with two of the geese dying at 5 dpi. Similar clinical signs were also observed in the contact geese starting on the 3^rd^ day post-contact (dpc). Of the contact geese four of the five died or were euthanized when moribund by 11 dpc. All five contact chickens died or were euthanized when moribund by 5 dpc. Gross lesions in the geese included conjunctivitis; pinpoint areas of hemorrhage on the myocardium, liver and abdominal fat; focal areas of necrosis on the pancreas; and hemorrhagic intestinal content with a hyperemic mucosal surface. The lungs were discolored and covered with a layer of fibrin and the air sacs were very thick. The female geese had egg yolk peritonitis. The spleens were enlarged and dark with a marbled appearance. Figure 4A shows the survival curves for Chinese geese that were infected with 10,000 PFU of FAV-002/H5N1 and contact Chinese geese and chickens that were allowed to co-mingle 24 hrs post-infection.

#### Muscovy ducks

The clinical signs in the Muscovy ducks were milder, but of a more protracted nature when compared with the geese. Clinical signs were first observed on the 3^rd^ day after infection and lasted for up to 14 days. Clinical signs ranged from depression and open mouth breathing to neurological signs in some of the animals. The neurological signs appeared after 7 dpi and included circling, head twitching and ataxia and most of these birds were able to recover from the disease. Of the fifteen inoculated ducks eleven showed clinical signs and four died or were euthanized. Two of the five contact ducks presented with clinical signs both of which died at 2 and 8 dpc respectively. All five contact chickens that were co-housed with the ducks to assess transmission died or were euthanized when moribund; the first died at 3 dpc and the last at 8 dpc. The major postmortem findings were pulmonary edema and fibrinous pleuritis in lungs and hyperemic tracheas. In some ducks, the spleens were dark, enlarged and had white necrotic areas. The pancreas exhibited large white patches of necrotic foci and the liver had multifocal small white areas of necrosis. Figure 4B illustrates the survival curves for Muscovy ducks that were infected with 10,000 PFU of FAV-002/H5N1 along with contact Muscovy ducks and chickens that were allowed to co-mingle beginning at 24 hrs post-infection.

#### Oral and cloacal shedding

**Figure 5:**
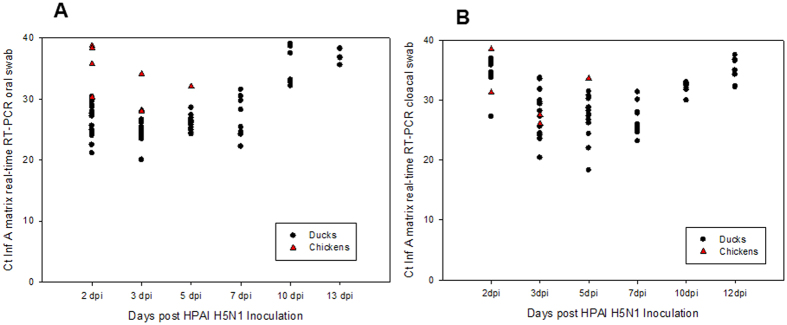
Virus shedding patterns of Muscovy ducks infected with 10,000 PFU of FAV-002/H5N1. Total RNA was extracted from oral (**A**) and cloacal (**B**) specimens and was tested using the avian influenza matrix based real time RT-PCR assay.

**Figure 6:**
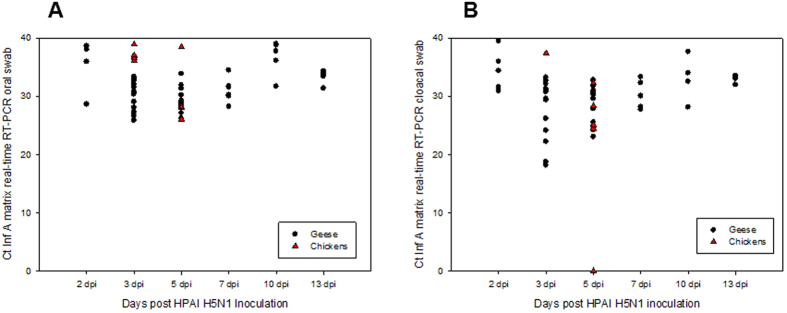
Virus shedding patterns of Chinese geese infected with 10,000 PFU of FAV-002/H5N1. Total RNA was extracted from oral (**A**) and cloacal (**B**) specimens and was tested using the avian influenza matrix based real time RT-PCR assay.

Virus shedding was assessed by influenza A virus matrix gene real-time RT-PCR assay on total RNA extracted from oropharyngeal and cloacal swab specimens. The shedding patterns of the infected ducks and contact chickens (Fig. 5) and geese and chickens (Fig. 6) were similar beginning as early as 2 dpi, reaching a peak by 3 to 5 dpi and persisting for up to 13 dpi. The magnitude of shedding in oropharyngeal (Figs 5A and 6A) and cloacal swab samples (Figs 5B and 6B) was similar.

#### Serology

**Figure 7:**
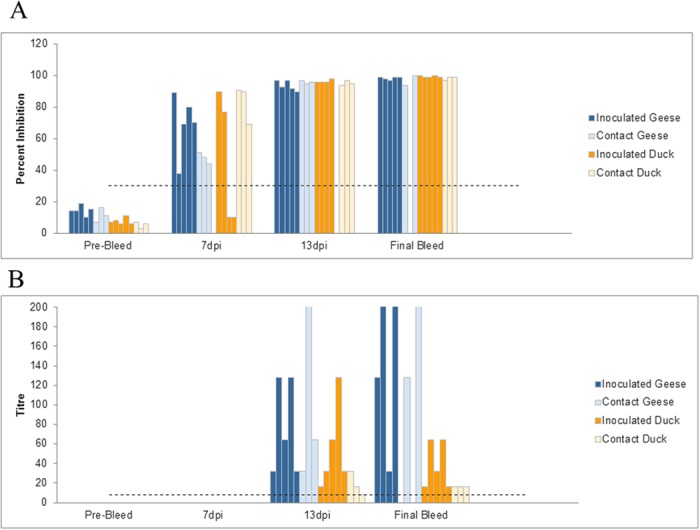
Serologic data for Chinese geese and Muscovy ducks that were infected with FAV-002/H5N1 virus and their corresponding contact groups. (**A**) shows influenza A virus nucleoprotein antibodies levels at different intervals of time using cELISA. Values above 30% inhibition (dotted line) were considered positive. No significant differences were found among inoculated versus contact ducks and geese at each time point. Significant differences (P value < 0.001) were however found between final bleed versus pre-bleed, 13 dpi versus pre-bleed, 7 dpi versus pre-bleed, final bleed versus 7 dpi and 13 dpi versus 7 dpi groups based on two-way ANOVA followed by the Holm-Šídák multiple comparison test. (**B**) illustrates anti-H5 antibody levels based at different intervals of time. Antibody detection is based on the hemagglutination-inhibition test using homologous FAV-002/H5N1 antigen. HI titers above 8 (dotted line) were considered positive. A statistically significant (P = 0.023) interaction was found between animal groups (inoculated/contact geese and inoculated/contact ducks) and time post-inoculation. Statistically significant differences were observed in HI titers of animals between the following time points: final bleed versus pre-bleed (P < 0.001), final bleed versus 7 dpi (P < 0.001), 13 dpi versus pre-bleed (P = 0.027) and 13 dpi versus 7 dpi (P = 0.022). Inoculated geese showed significantly different HI titers at the following time points: final bleed versus pre-bleed (P < 0.001), final bleed versus 7 dpi (P < 0.001) and final bleed versus 13 dpi (P = 0.003). Finally significant differences in HI titers were observed in the contact geese group at the following time points: final bleed versus pre-bleed (P = 0.011) and final bleed versus 7 dpi (P = 0.013). Analysis was by two-way ANOVA followed by the Holm-Šídák multiple comparison test.

Chinese geese and Muscovy ducks that were infected with FAV-002/H5N1 virus began to develop influenza A virus nucleoprotein antibodies at 7 dpi with cELISA percent inhibition values peaking by 13 dpi (Fig. 7A). H5 antibodies were first detected at 13 dpi and peaked at the termination of the experiment (Fig. 7B).

#### Microscopic lesions in birds

**Figure 8:**
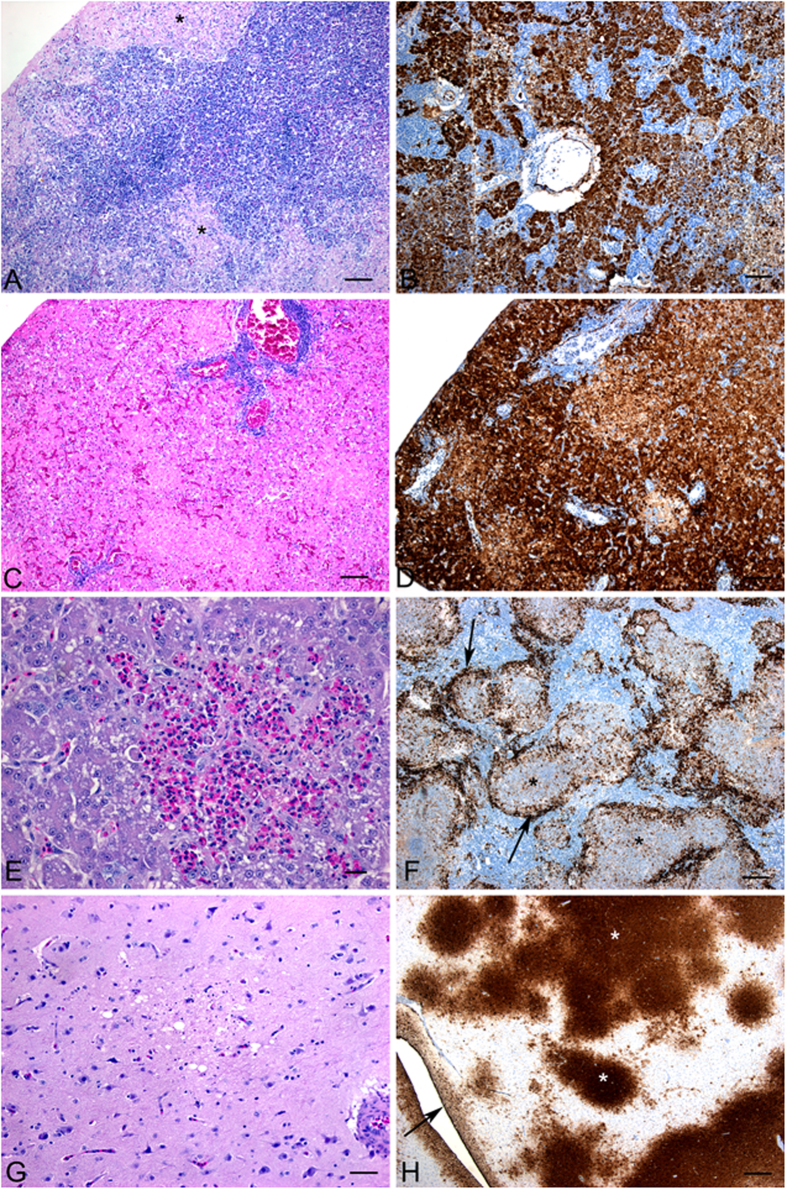
Microscopic lesions observed in geese, turkeys and ducks that were infected with FAV-002/H5N1. (**A**) Extensive necrosis in the pancreas of a Chinese goose. (**B**) Presence of abundant viral antigen in the pancreas of a Chinese goose. (**C**) Presence of large areas hepatic necrosis with heterophil infiltration in a turkey. (**D**) Extensive area of hepatic necrosis associated with positive immuno-staining (arrow) in a goose. (**E**) Large multifocal areas of necrosis in the spleen of a turkey. (**F**) Positive immuno-staining in the multifocal areas of necrosis in the spleen of a Chinese goose. (**G**) Brain lesions in Muscovy ducks were subtle and variable and included necrosis with gliosis and meningitis, ependymitis and inflammation of the choroid plexus. (**H**) Extensive viral antigen (*) in the brain of the turkey.

The histological lesions observed in geese, turkeys and ducks were similar with some variations described below. The lesions are illustrated in Fig. 8A–H. In all species there were areas of extensive pancreatic necrosis. This was associated with abundant viral antigen in geese and turkeys but smaller amounts of antigen in the ducks. Significant hepatic necrosis was observed in all species and varied from massive hepatic necrosis with associated extensive positive immunostaining in the geese, to multifocal lytic necrosis with heterophil infiltration in the turkeys. Splenic necrosis was observed in all species with varying severity and was associated with immunopositivity for viral antigen. Brain lesions were subtle and variable and included necrosis with gliosis as well as meningitis, ependymitis and inflammation of the choroid plexus. Extensive viral antigen was detected in the brain of the turkeys while moderate amounts were observed in the geese and ducks. Lesions observed in the cecal tonsil included hemorrhage, lymphoid depletion and inflammation. Table 2 summarizes the relationship between viral antigen load as determined by immunostaining and influenza A matrix gene copy number as determined indirectly by real-time RT-PCR assay for various tissues in inoculated and contact birds.

### Pathobiological characteristics of FAV-002/H5N1 in mice and pigs

#### Mice

**Figure 9:**
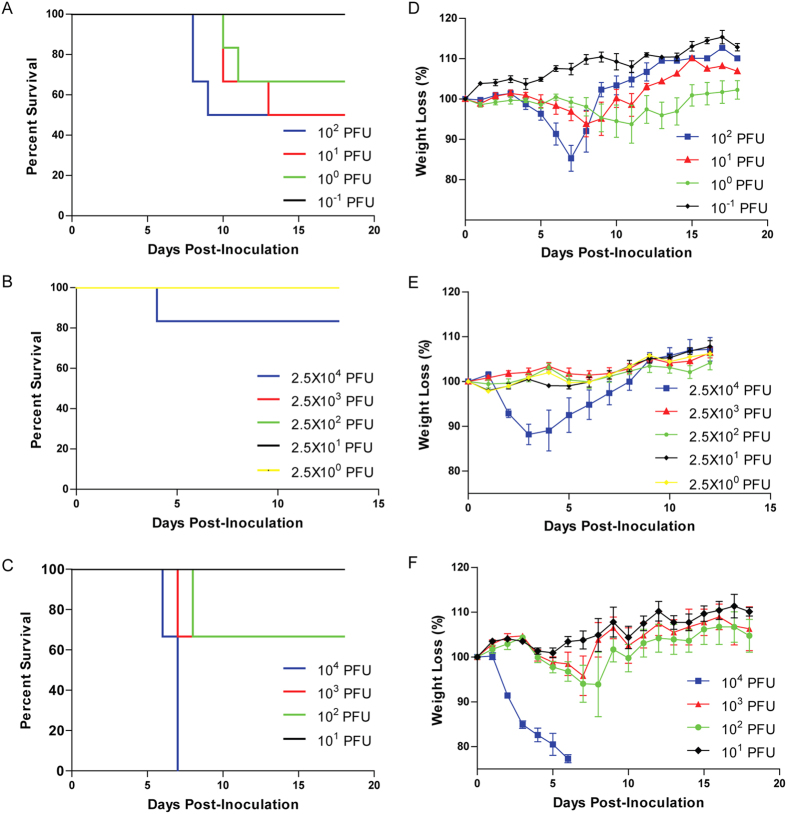
Survival and weight loss of mice infected with H5N1 and H5N8 viruses. Six- to 7-week-old female Balb/c mice were intranasally challenged with 10 fold serial dilutions of FAV-002/H5N1 (**A**,**D**), AGT/H5N1 (**B**,**E**) and AW-050/H5N8 (**C**,**F**) and observed for survival and weight loss. The percent survival was determined and mouse 50% lethal dose (MLD50) was calculated by the Miller and Tainter method (43). N = 6 mice per group.

FAV-002/H5N1 was found to be highly virulent in mice with an LD_50_ of 46 PFU. In contrast, the A/American wigeon/BC/050-31/2015 (H5N8) virus (designated AW-050/H5N8) was moderately pathogenic to mice with an LD_50_ of approximately 776 PFUs and the A/American green-winged teal/Washington/ 195750/2014 (H5N1) virus (designated AGT/H5N1) was mildly pathogenic with an LD_50_ > 2.5 × 10^4^ PUUs. Survival curves of FAV-002/H5N1, AGT/H5N1 and AW-050/H5N8 inoculated mice are shown in Fig. 9A–C respectively. Weight loss was dose dependent in the FAV-002/H5N1 (Fig. 9D), AGT/H5N1 (Fig. 9E) and AW-050/H5N8 (Fig. 9F) groups. Mice inoculated with 10^4^ PFUs of AW-050/H5N8 exhibited a significant weight loss of approximately 25% body weight by 6 dpi, in contrast to the mice in the group that received lower amounts of virus which exhibited <10% weight loss at 3–7 dpi (Fig. 9F). To determine the pathogenicity of FAV-002/H5N1, five mice were infected with 10^2^ PFU of virus and sacrificed at 3 and 6 dpi, and lungs, heart, gut, kidney and spleen were harvested for RNA extraction. Viral RNA was found in the lungs of the mice at 3 and 6 dpi, having log_10_ values of 4.0 and 5.7 copies/gram of tissue respectively. In addition, low copies of viral RNA (3.66 × 10^1^ and 7.11 × 10^1^ copies/gram of tissue) were detected in the kidney and heart of only one mouse at 6 dpi. Viral RNA was not detected in the gut or spleen of any of the other animals.

**Figure 10:**
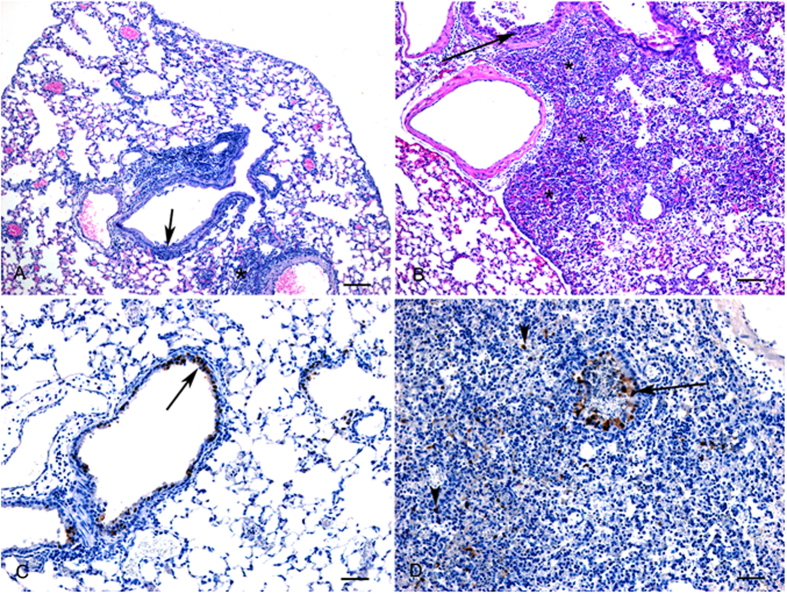
Histopathology (HE) and immunohistochemistry (IHC) findings in lungs from H5N1 infected mice. (**A**) Day 3: Mild to moderate bronchiolitis (arrow) and scattered foci of interstitial pneumonia (*). HE, bar = 100 μm. (**B**) Day 6: Large areas of necrotizing interstitial pneumonia (*) and bronchiolitis (arrow). HE, bar = 100 μm. (**C**) Day 3: Positive immunostaining for influenza A antigen is primarily observed in bronchiolar epithelial cells (arrow). IHC, bar = 50 μm. (**D**) Day 6: Viral antigen is detected in bronchiolar epithelial cells (arrows) as well as in macrophages (arrowheads). IHC, bar = 50 μm.

Histologic lesions in mouse lungs were mild at 3 dpi and characterized by necrosis and inflammation in occasional bronchioles and a few small foci of necrotizing interstitial pneumonia (Fig. 10A). At 6 dpi, the lesions were similar, but were more extensive involving up to 30% of the examined lung section (Fig. 10B) with the addition of vasculitis. Influenza A viral antigen could be detected in bronchiolar epithelial cells at both time points (Fig. 10C). At 6 dpi detection of viral antigen was frequently detected in interstitial cells which appeared morphologically to be macrophages (Fig. 10D).

#### Pigs

**Figure 11:**
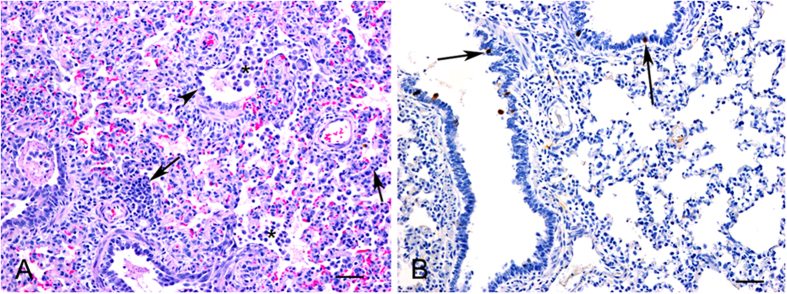
Histopathology (HE) and immunohistochemistry (IHC) findings in lungs from FAV-002/H5N1 infected pigs. (**A**) Microscopic lesions in a lung characterized by interstitial infiltration of inflammatory cells (arrows), mild bronchiolar changes with degeneration and disorganization of epithelial cells (arrowhead) and increased numbers of macrophages in bronchiolar lumens and alveoli. HE, bar = 50 μm. (**B**) Positive immunostaining for influenza A viral antigen was detected in scattered bronchiolar epithelial cells. IHC, bar = 50 μm.

No clinical signs were observed in any of the pigs following intranasal inoculation with FAV-002/H5N1. No detectable levels of influenza A virus RNA (Ct values < 35) were found by real-time RT-PCR in all oral and nasal swabs collected at different time points with the exception of one nasal swab collected from a single pig at 1 dpi. Histologic lesions were observed in some of the lung tissues at both 3 and 5 dpi and were characterized by expansion of alveolar walls due to inflammatory cell infiltration, mild edema and increase in numbers of alveolar macrophages consistent with mild to moderate interstitial pneumonia (Fig. 11). Bronchiolar changes were mild consisting of some disorganization and degeneration of epithelial cells (Fig. 11A). In addition, in some areas there was perivascular and peribronchiolar cuffing of mononuclear inflammatory cells. Limited influenza viral antigen was detected in occasional bronchiolar epithelial cells (Fig. 11B) as well as rare cells within alveolar walls morphologically consistent with macrophages. Positive immunostaining was also observed in rare tracheal epithelial cells. The two pigs that did not undergo timed postmortem examinations did develop influenza A virus nucleoprotein antibodies as determined by cELISA.

#### Neuraminidase Enzyme Kinetics

**Figure 12:**
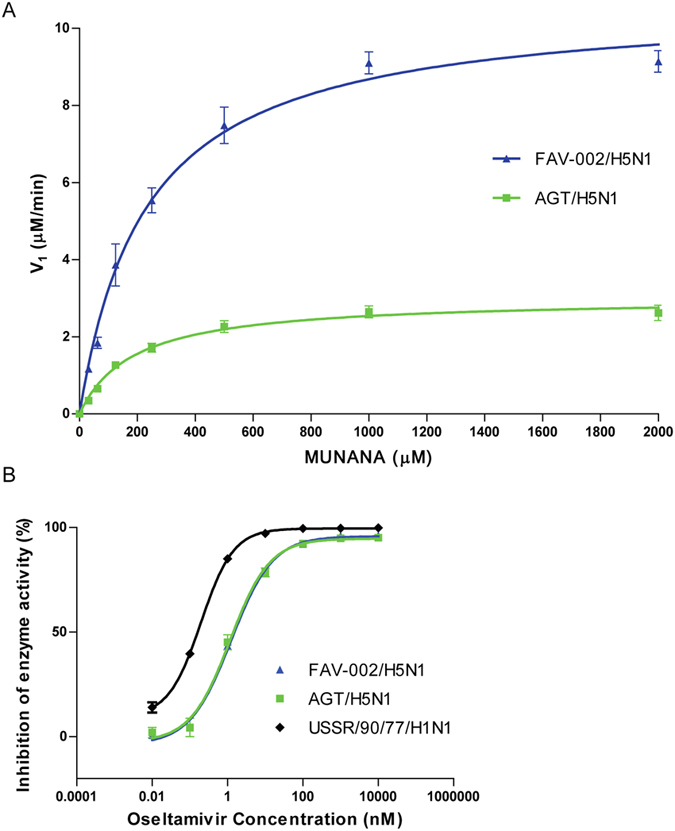
Neuraminidase enzyme kinetics and sensitivity of H5N1 viruses to oseltamivir. (**A**) Neuraminidase kinetic curves for FAV-002/H5N1 and AGT/H5N1. Relative fluorescent unit (RFU) values for each virus (FAV-002/H5N1 and AGT/H5N1) were plotted against a 2-fold dilution series of MUNANA to determine the maximum velocity (Vmax) and the binding affinity (Km) to MUNANA for each virus. (**B**) Comparative sensitivity to oseltamivir for each virus is reflected by the percent inhibition of enzyme activity at each concentration of oseltamivir carboxylate, plotted as the ratio of RFU at each dose over the RFU value with no drug treatment. P value < 0.0001.

Since the major difference between FAV-002/H5N1 and AGT/H5N1 was the length of the NA gene, it was important to determine whether the viruses exhibit any difference in NA activity. Comparison of the NA activities of FAV-002/H5N1 and AGT/H5N1 demonstrated that the Km value was not significantly different between the two viruses (Km FAV-002/H5N1 = 230.8 ± 25.21 and AGT/H5N1 186.9 ± 24.37). However, there was more than a three-fold increase in the V_max_ of FAV-002/H5N1 (V_max_ = 10.68 relative fluorescent units/sec ± 0.3638; p value < 0.0001) compared to AGT/H5N1 (V_max_ = 3.01 relative fluorescent units/sec ± 0.1160; p value < 0.0001). NA enzyme kinetic results are illustrated in Fig. 12A.

#### Oseltamivir Sensitivity

FAV-002/H5N1 and AGT/H5N1 are both susceptible to oseltamivir with IC_50_ concentrations of 1.259 (range 0.8421–1.868) and 1.170 (range 0.8549–1.601) nM respectively. A/USSR/90/1977 (H1N1) was used as a control and had an IC_50_ of 0.1986 (range 0.1725 and 0.2287) nM. Results of this experiment are summarized graphically in Fig. 12B.

## Discussion

The unprecedented transcontinental movement of Eurasian H5N8 virus to the west coast of North America was the priming event that led to the creation of two novel reassortant H5N1 and H5N2 viruses. The North American gene segments that comprise the reassortant H5N1 and H5N2 viruses appear to have originated from different wild bird origin progenitor viruses. According to the APHIS website (http://www.aphis.usda.gov), three novel reassortant H5N1 viruses including AGT/H5N1 were isolated in Whatcom County, Washington; two from American wigeons and one from an American green winged teal. The novel reassortant FAV-002/H5N1 described in the current study was isolated from a backyard chicken flock in the Fraser Valley of British Columbia. FAV-002/H5N1 was almost identical to the isolate from the US AGT/H5N1 except for a 19 amino acid deletion in the stalk of the neuraminidase in addition to a number of other minor changes involving other gene segments. NA stalk deletions have been associated with the adaption of virus from natural waterfowl reservoir hosts to gallinaceous species as well as with broader host range and virulence^22^. We were not able to detect the presence of untruncated NA similar to that seen in AGT/H5N1 from the backyard flock where FAV-002/H5N1 was isolated. This might indicate that the deletion occurred at the very early stages of infection before sampling was conducted, or the truncation may have already existed in the viral population before it was introduced to the backyard flock. Previous studies^23^,^24^ have demonstrated that NA stalk deletions are often accompanied by the addition of glycosylation sites in the HA protein which is thought to be necessary to maintain a functional balance between the HA and NA, the latter being an essential requirement for infectivity. Notably, no corresponding changes were found in the HA of FAV-002/H5N1. The NA stalk deletion found in FAV-002/H5N1versus AGT/H5N1 was associated with a more than three-fold increase in Vmax but no significant change in substrate affinity. This is in contrast to the decreased NA activity of influenza A viruses with truncated NA stalks that has been reported by others^25^,^26^,^27^. This decrease in NA activity, expressed as diminished ability of the virus to elute from red blood cells, is thought to be due to the NA not being able to access its sialic acid substrate properly, possibly because the enzyme active site is located too close to the viral envelope. Obviously the functional balance between HA and NA is complex and any advantage provided by NA stalk deletions may be dependent on several factors including the host and specific HA-NA pairings. Noteworthy is the fact that all Gs/Gd-lineage H5N1 viruses that spread from Asia to other regions possessed NA stalk deletions that were similar to the one found in FAV-002/H5N1^28^.

Since the emergence of the Gs/Gd-like H5N1 viruses in 1996, the length of the neuraminidase stalk region has varied considerably among different strains and this is thought to be associated with virulence in some animal hosts^29^. According to Matsuka *et al*.^29^, Eurasian H5N1 viruses with a truncated NA stalk possessed an increased virulence for mice but not chickens when compared with viruses with an untruncated stalk. Viruses with NA stalk deletions were 10,000-fold more pathogenic than their counterpart H5N1 viruses with no deletion. In the current study, the mouse LD_50_ value for the novel FAV-002/H5N1 virus was low (46 PFU) and the value was almost identical to pathogenic strains of Gs/Gd-like HPAI H5N1 viruses indicating some similarity in terms of virulence in mice. In contrast, the LD_50_s of the AGT/H5N1 virus without a truncated NA stalk, and the AW-050/H5N8 virus were both significantly higher. These results are consistent with previous findings that H5N1 viruses with truncated NA stalks are more virulent for mice and that clade 2.3.4.4 H5N8 viruses produce mild clinical disease in experimentally infected mice, dogs and ferrets^6^,^21^.

The PB1-F2 of FAV-002/H5N1 encodes for a protein of 90 AA and has serine at position 66 that was previously demonstrated to impact virulence in Eurasian HPAI H5N1 viruses by inhibiting interferon induction through binding of the PB1-F2 to the MAVS adaptor protein^30^. In addition, the PB1-F2 has some additional molecular signatures (62L, 75R, 79R, and 82L) that are known to contribute in the cytokine release and inflammatory responses^30^. None of the remaining mutations were found to be associated with pathogenicity based on inventory of amino acid mutations in H5N1 viruses used by the influenza surveillance and research community as a tool to inform the influenza knowledge base for surveillance and public health preparedness. http://www.cdc.gov/flu/pdf/avianflu/h5n1-inventory.pdf.

Hemagglutinin protein sequences of the novel FAV-002/H5N1, AGT/H5N1 and AW-050/H5N8 were identical at their respective receptor binding sites. Consequently, FAV-002/H5N1 should have similar binding preference for avian α2–3 receptors and to a lesser extent α2–6 receptors indicating inadequate zoonotic potential. Despite this it should be noted that recent human infections involving clade 2.3.4.4 H5N6 viruses in China have resulted in severe respiratory disease and even death^31^,^32^. During the mammalian animal trials, pigs that were infected with FAV-002/H5N1 were sub-clinically affected but did seroconvert. Virus shedding was very minimal and microscopically the lesions were restricted to the lungs and were very mild with only a small number of lung cells positive for influenza A virus antigen as determined by IHC. Previous experimental studies in pigs using various Gs/Gd-like H5N1 HPAI viruses resulted in either no virological or serological confirmation of infection or mild to moderate infection that was restricted to the respiratory tract^33^,^34^.

Experimental studies in mice with Gs/Gd-like HPAI H5N1 have demonstrated that viruses which exhibited high virulence in mice replicated in the lungs and other organs without prior adaptation, while viruses of low virulence were restricted to the respiratory tract^21^,^27^,^29^. In the current study, FAV-002/H5N1 was able to replicate in the lungs of mice causing necrotizing interstitial pneumonia and vasculitis. Presence of viral antigen in the lungs was also confirmed by IHC and by real-time RT-PCR; however, despite the highly pathogenic nature of the FAV-002/H5N1, only minimal levels of viral nucleic acid were detected in the heart and kidneys of one mouse at 6 dpi. This lack of dissemination may be due in part to the absence of E627K and D701N mutations in PB2 which are associated with higher polymerase activity and improved viral replication in mammalian cells as well as enhanced virulence in mice^35^.

Historically, influenza A viruses of all existing HA and NA subtypes have been isolated from different Anseriformes and Charadriiformes genera and species and are not normally associated with clinical disease or mortality. This changed after the emergence of Gs/Gd-like H5N1 viruses in 1996 as various strains were capable of killing different species of ducks, geese and shorebirds^36^,^37^. Similar to Gs/Gd-like H5N1 viruses that were found to be highly pathogenic to waterfowl, FAV-002/H5N1 was also pathogenic in experimentally infected Chinese geese (*Anser cynoides domesticus*) and Muscovy ducks (*Cairnia moschata foma domestica*) and was able to replicate systemically in both species. The virus was shed cloacally and orally and transmitted to and caused mortality in naïve contact chickens, ducks and geese. To date there is no information that FAV-002/H5N1, and its closest ancestor AGT/H5N1 that was isolated from hunter killed green-winged teal (*Anas crecca*), have been associated with any die-offs or clinical disease in wild waterfowl. So far this novel reassortant virus has only been detected in the lower Fraser Valley of British Columbia and in Whatcom County, Washington. Both are located within the Pacific flyway and there is no evidence of spread to other flyways. Furthermore, there is no indication thus far that this virus has been associated with any additional outbreaks in gallinaceous species.

## Methods

### Case Report

In February 2015, a backyard chicken layer flock consisting of 100 birds near Chilliwack, British Columbia experienced a sudden increase in mortality. Swab and tissue specimens collected from this flock were submitted to the provincial veterinary diagnostic laboratory (Abbotsford, BC). The samples tested positive using modified versions of the Spackman *et al*.^38^ influenza A matrix gene and H5 hemagglutinin gene real-time RT-PCR assays and were sent to the National Center for Foreign Animal Disease Laboratory (NCFAD) in Winnipeg, Manitoba for confirmation. Twelve tissue specimens collected in parallel by a Canadian Food Inspection Agency (CFIA) team were also sent to NCFAD for analysis.

### Viruses

A/chicken/BC/FAV-002/2015 (H5N1) (hereafter designated FAV-002/H5N1) was isolated from a backyard layer chicken flock near Chilliwack, British Columbia. The full genome sequence of FAV-002/H5N1 has been deposited in the NCBI data base with accession numbers KP892988 to KP892995. A/American wigeon/BC/050-31/2015 (H5N8) (hereafter designated as AW-050/H5N8) was isolated from a hunter killed American wigeon in our laboratory. The virus was isolated in embryonating specific pathogen free (SPF) chicken eggs and the virus sequences are deposited in GISAID website under accession number EPI_ISL_178249. A/American green-winged teal/Washington/ 195750/2014 (H5N1) (hereafter designated as AGT/H5N1) was kindly provided to us by Dr M.K. Torchetti (Diagnostic Virology Laboratory, National Veterinary Services Laboratories, Ames, Iowa). The sequence of this virus is available at NCBI data base under accession numbers KP739418 to KP739425.

### Virus isolation and characterization

Swab and tissue specimens that screened positive on AIV matrix and H5 real-time RT-PCR assays were used for virus isolation by inoculating the allantoic cavity of 9-day-old SPF embryonated chicken eggs (ECE). The presence of virus in the allantoic fluids harvested from dead embryos was confirmed by hemagglutination assay and the agent was typed by hemagglutination-inhibition assay using standard methods (http://www.oie.int/fileadmin/Home/eng/Health_standards/tahm/2.03.04_AI.pdf_AI.pdf). In addition, total RNA was extracted from allantoic fluids and the presence of AIV confirmed by influenza A matrix and H5 based real-time RT-PCR assays.

### Virus genome sequencing

#### Sanger Sequencing

Total RNA was extracted directly from field specimens and from allantoic fluids using the MagMax™- 96 AI/ND Viral Extraction Kit (Ambion) and MagMax Express robotic system according to the manufacturer’s recommendations (Life Technologies). The eight influenza A viral gene segments were amplified in one step RT-PCR using a universal primer set^39^ and the qScript XLT One-Step RT-PCR Kit (Quanta Biosciences). The amplified RT-PCR products were resolved by agarose gel electrophoresis, cut from the gel and purified using a QIAquick gel extraction kit (Qiagen). The purified RT-PCR amplicons were then ligated into pCR4-Topo (Life Technologies) and used to transform OneShot competent *E. coli*. The resulting plasmids were sequenced using BigDye Terminator chemistry version 3.1 (Life Technologies) and an Applied Biosystems 3130xl Genetic Analyzer.

#### Sequencing using Ion Torrent

Full-length influenza A virus gene segments were amplified directly from total RNA that was extracted from 10% (w/v) homogenates made from clean (brain, lungs, spleen and heart) and dirty (gut and its contents) tissue pools following the protocol described by Hoffman *et al*.^39^. In addition, the NA genes were amplified from RNA extracted from all swab and tissue specimens collected from different chickens. The RT-PCR products were purified as described above. Purified gene segments were subsequently quantified using a Biodrop Touch UV spectrophotometer. Equimolar pools of all gene segments were generated when performing whole genome sequencing. Library construction was performed using the Ion Xpress™ Plus Fragment Library Kit (Life Technologies) utilizing a 5 minute shearing time. IonXpress™ Barcode adapters (Life Technologies) were applied to each full genome isolate or individual gene segments when required. Size selection of each barcoded library was performed on a PIPPIN-Prep using 2% agarose gels (Sage Science). Size-selected libraries were qualitatively assessed using the Agilent High Sensitivity DNA Kit and Agilent 2100 Bioanalyzer. qPCR was performed with a 7500 Fast Real-Time PCR System (Applied Biosystems) and the Ion Library Quantitation Kit (Life Technologies) to determine template dilution factor for emulsion PCR. Barcoded libraries were pooled and DNA template was prepared for sequencing using the Ion PGM™ Template OT2 Reactions 200 kit (Life Technologies) with the Ion OneTouch™2 System with ES for ISP enrichment. Quality control of Ion Sphere Particles (ISP) was performed using Ion Sphere™ Quality Control Kit and a Qubit fluorometer (Life Technologies). Sequencing was performed with an Ion Torrent PGM™ instrument using an Ion 314™ Chip Kit v2 (Life Technologies) and an Ion PGM ™Hi-Q™ Sequencing Kit. Data analysis was performed using DNAstar SeqMan NGen® software, DNAstar Core suite and BLAST server at NCBI. During library build, Agencourt AMPure XP Reagent (Beckman Coulter, Inc.), was used for purification when required following all manufacturer protocols.

#### Phylogenetic analysis

Viral gene sequences were aligned with other sequences available on public databases (NCBI and GIAID data bases) using ClustalW and evolutionary history was inferred by using the Maximum Likelihood method based on the Tamura-Nei model^40^. The number of bootstrap replications was set to 1000, and major tree branches were labeled with bootstrap values >75%. The analysis was performed using MEGA version 6 software^41^.

#### Animal Experiments

All experiments on live vertebrates were carried out in accordance with guidelines set out by the Canadian Council on Animal Care and all experimental manipulations carried out on live animals were approved by the Canadian Science Centre for Human & Animal Health Animal Care Committee.

Chinese Geese and Muscovy ducks: Twenty Chinese geese (*Anser cynoides domesticus*) were purchased from a local supplier in Manitoba and 20 Muscovy ducks (*Cairnia moschata foma domestica*) were purchased from a private farm in Ontario. The geese and ducks were transported to the National Centre for Foreign Animal Disease (NCFAD) and were housed in biosafety level 3 plus (BSL-3+) animal cubicles. The geese were provided *ad-libitum* water and fed with Goose Grower from Master-feeds grain. Dried corn was given as supplement. The birds were floor housed and had access to a wading pool and filled water troughs.

Chickens and turkeys: Twenty 4- to 6-week-old Leghorn chickens (*Gallus gallus domesticus*) were acquired from the CFIA SPF flock in Nepean, Ottawa. Twenty 8- to 10-week-old AIV free turkey (*Melleagris gallopavo*) pullets were purchased from a local supplier in Manitoba. The chickens and turkeys were placed in BSL-3+ animal cubicles. The chickens and turkeys were fed with 20% Chick Starter feed from Masterfeeds and water provided *ad libitum*. Heat lamps provided as source of supplemental heat. Perches were provided as environmental enrichment.

### Intravenous pathogenicity index (IVPI)

For this purpose, ten 4- to 6-week-old SPF Leghorn chickens were inoculated intravenously with 0.1 ml of 1:10 dilution of sterile allantoic fluid containing FAV-002/H5N1. Ten control chickens were mock inoculated with the PBS diluent. Scores were collected according to procedures described in the OIE Manual of Diagnostic Tests and Vaccines for Terrestrial Animals (http://www.oie.int/international-standard-setting/terrestrial-manual/access-online/).

### Mean chicken lethal dose (CLD_50_)

Six-week-old SPF Leghorn chickens were placed in 4 different animal cubicles. Three groups of five chickens were inoculated intranasally with different dilutions of FAV-002/H5N1. The 4^th^ group was mock inoculated with PBS. The birds were observed daily for clinical signs and mortality over a period of 2 weeks.

### Pathogenesis studies in different species of birds

#### Ducks and Geese

After a week of acclimatization, 15 Chinese geese and 15 Muscovy ducks, each housed in separate animal cubicles, were inoculated with 10^4^ PFU of FAV-002/H5N1 by oronasal and cloacal routes. Twenty-four hrs following inoculation, 5 naïve Chinese geese and 5 naïve Muscovy ducks were placed amongst the corresponding groups to commingle and assess virus transmission. In addition, 5 naïve chickens housed in wire dog kennels were placed in each corner of the animal cubicle with infected Chinese geese and Muscovy ducks. The cages housing the chickens were placed so that a portion of each cage had contact with the standing water in the animal cubicle. Swabs were collected from all birds on 2, 3, 5, 7, 9, 14 and 20 days after infection or contact.

#### Turkeys

After a week of acclimatization, 10 turkeys were inoculated intranasally with 10^3^ PFUs of FAV-002/H5N1. Twenty-four hrs following inoculation, 5 naïve contact turkeys were allowed to commingle amongst the infected turkeys to assess virus transmission. Swabs were collected on 2, 3 and 5 dpi.

### Pathogenesis studies in mammals

#### Pigs

Four- to six-week-old Duroc piglets were obtained from a local supplier in Manitoba and housed in the BSL-3+ animal cubicles at NCFAD. The piglets were acclimatized for 1 week and blood and nasal swabs collected prior to virus inoculation. Piglets tested negative for influenza A virus nucleoprotein antibodies as determined by cELISA^42^ and for influenza A virus RNA as determined by real-time RT-PCR (31). Seven piglets were challenged by intranasal administration of 10^6^ 50% tissue culture infectious doses (TCID_50_) of H5N1 in a 1 ml volume. Sera were collected from the piglets prior to infection as well as 14 and 21 days following infection. Oral and nasal swabs were taken prior to inoculation and every day for six days post-inoculation (dpi) and one piglet was euthanized on day 3, 4, 5, and 6 post- inoculation. Lungs specimens including the apical, cardiac and caudal lobes were collected; half of the specimens were fixed in 10% formalin and the other half were frozen at −70 °C.

#### Mice

Female Balb/c mice, 6–7 weeks old (Charles River Laboratories, QC) were used for all experiments. To determine the mouse LD_50_ (MLD_50_), groups of six mice were inoculated with 50 μl of virus per mouse intranasally with 10-fold dilutions of FAV-002/H5N1, AGT/H5N1 and AW-050/H5N8 viruses. MLD_50_ was calculated by the Miller and Tainter method^43^. To determine the pathogenicity of the virus five mice were infected with 10^2^ PFU of virus and sacrificed at 3 and 6 days post infection. Lungs, heart, gut, kidney and spleen were harvested for RNA detection using RNAlater (Qiagen, ON, Canada) to preserve the RNA, followed by RNA extraction using the RNeasy Plus Mini kit (Qiagen). qRT-PCR was performed using the Lightcycler 480 RNA Master Hydrolysis Probe kit (Roche, ON, Canada) along with primers and probes targeting the matrix protein of influenza^38^. RNA was quantified by comparison to a standard curve based on known concentrations of plasmid (pPol vector) containing the H5N1 virus matrix gene.

#### Real-time RT-PCR assay

The presence of influenza A virus in swabs and tissues collected from animals was carried out using a semi quantitative real-time RT-PCR (RRT-PCR) assay described by Spackman *et al*.^38^. Full length*, in vitro* transcribed segment 7 RNA, serially diluted in RNase free water was run with each assay in order to give a semi-quantitative estimate of the viral load in each sample.

#### Neuraminidase Enzyme Kinetics

Virus titres were determined by plaque assay on MDCK cells (ATCC, VA, USA). Following adsorption of virus in 10-fold serial dilutions, cells were overlayed with a 1% SeaPlaque® Agarose (Lonza, ME, USA) mixture containing minimal essential medium (MEM) and 1μg/ml TPCK-trypsin. For the enzymatic assay, the substrate 2′-(4-Methylumbelliferyl)-a-D-N-acetylneuraminic acid (MUNANA; Sigma, ON, Canada) was diluted in a 2-fold series (2000 μM to 0 μM) and 10 μl was added into 96 well white round bottom plates (Grenier Bio-One, NC, USA). Virus was adjusted to 10^5^ PFU/ml and 10 μl added to each well. The plate was read in a Synergy HTX plate reader (BioTek, VT, USA) pre-warmed to 37 °C. Fluorescence was captured every 90 seconds at 37 °C for 60 minutes using excitation at 360 nm and emission at 460 nm. Data at each dilution of MUNANA was analyzed for linear regression and the rate of increase in fluorescence was determined for each dilution. This data was then analyzed with GraphPad Prism (GraphPad Software, CA, USA) by nonlinear regression (Enzyme Kinetics – Michaelis Menten) to determine the Vm and Kmax for each virus.

#### Oseltamivir Sensitivity

Based on the Neuraminidase Enzyme Kinetics data we determined which combination of virus and MUNANA substrate would result in a signal of approximately 2000 Relative Fluorescence Units (RFU) following 30 minutes of incubation. This required 3.3 × 10^4^ PFU/ml of FAV-002/H5N1 and 1 × 10^4^ PFU/ml of AGT/H5N1 along with 667 μM of MUNANA. Oseltamivir carboxylate (Toronto Research Chemicals, ON, Canada) was diluted in a 10-fold series (10 μM to 0.01 nM) and each corresponding virus was added to each dilution and incubated for 30 minutes at 37 °C. The virus/oseltamivir mixture was then added to a round bottom plate containing MUNANA, incubated at 37 °C for 30 minutes and read using excitation at 360 nm and emission at 460 nm. Inhibition of enzymatic activity values were then calculated as the percent reduction in RFU relative to control wells containing no oseltamivir.

#### Postmortem examinations

For birds, timed postmortem examinations were performed on 3, 5 and 7 dpi. In addition, postmortem examinations were conducted on moribund birds. Birds were euthanized by exsanguination after being anaesthetized with isofluorane. Samples that included brain, spinal cord, lung, liver, spleen, cecal tonsil, esophagus, trachea, pectoral muscle, kidney, heart, nasal turbinate, pancreas, duodenum, ileum, proventriculus, and thymus were collected. For pigs, timed postmortem examinations were conducted on days 3, 4, 5 and 6 post-inoculation, and tissues that included apical, cardiac and caudal lung lobes were collected. For mice, timed postmortem examinations were performed at 3 and 6 dpi and lungs, heart, gut, kidney and spleen were collected. For all species, collected tissues were split and preserved for molecular and microscopic analysis. All tissues collected for microscopic analysis were fixed in 10% neutral buffered formalin and routinely processed for histopathologic examination. For immunohistochemistry, paraffin tissue sections were quenched for 10 minutes in aqueous 3% H_2_O_2_ then pretreated with proteinase K for 15 minutes. The primary antibody was a mouse monoclonal antibody F26NP9^42^ specific for influenza A nucleoprotein (NP) and was used at a 1:10,000 dilution for one hour. They were then visualized using a horseradish peroxidase labelled polymer, Envision® + system (anti-mouse) (Dako, USA), reacted with the chromogen diaminobenzidine (DAB). The sections were then counter stained with Gill’s hematoxylin.

## Additional Information

**How to cite this article**: Berhane, Y. *et al*. Pathobiological Characterization of a Novel Reassortant Highly Pathogenic H5N1 Virus Isolated in British Columbia, Canada, 2015. *Sci. Rep.* **6**, 23380; doi: 10.1038/srep23380 (2016).

## Figures and Tables

**Table 1:** Percent nucleotide and protein identity between FAV-002/H5N1 and top NCBI BLAST matches for eight gene segments.

**Segment/Lineage**	**% Nucleotide Identity similarity**	**% Protein identity similarity**
PB2/Eurasian	A/gyrfalcon/Washington/41088-6/2014(H5N8) 2276/2280 (99%)	A/gyrfalcon/Washington/41088-6/2014(H5N8) 758/759 (100%)
PB1/North American	A/American green-winged teal/Washington/195750/2014(H5N1) 2270/2274 (99%)	A/American green-winged teal/Washington/195750/2014(H5N1) 756/757 (99%)
PA/North American	A/American green-winged teal/Washington/195750/2014(H5N1) 2147/2151 (99%)	A/mallard/Alaska/44430-148/2008(H4N6) 715/716 (99%)
HA/Eurasian	A/American green-winged teal/Washington/195750/2014(H5N1) 1700/1704 (99%)	A/gyrfalcon/Washington/41088-6/2014(H5N8) 566/567 (99%)
NP/Eurasian	A/American green-winged teal/Washington/195750/2014(H5N1) 1496/1497 (99%)	A/American green-winged teal/Washington/195750/2014(H5N1) 498/498 (100%)
NA/North American	A/American green-winged teal/Washington/195750/2014(H5N1) 1312/1314 (99%)*	A/American green-winged teal/Washington/195750/2014(H5N1) 463/485 (96%)
M/ Eurasian	A/American green-winged teal/Washington/195750/2014(H5N1) 987/987 (100%)	A/American green-winged teal/Washington/195750/2014(H5N1) M1 - 97/97 (100%) & M2 - 252/252 (100%)
NS/North American	A/American green-winged teal/Washington/195750/2014(H5N1) 850/850 (100%)	A/American green-winged teal/Washington/195750/2014(H5N1) NS2 - 121/121 (100%); NS1 - 230/230 (100%)

*Excluding the 57 nucleotide deletion in the stalk region.

**Table 2:** Virus load as determined by IHC and RT-qPCR in tissues of inoculated and contact birds.

**Tissue**	**Geese Group**						**Duck Group**					
	**Inoculated goose 5 dpi**		**Contact goose 6 dpc**		**Contact chicken 4 dpc**		**Inoculated duck 5 dpi**		**Contact duck 8 dpc**		**Contact chicken 8 dpc**	
	**1Ct value**	**IHC**	**Ct value**	**IHC**	**Ct value**	**IHC**	**Ct value**	**IHC**	**Ct value**	**IHC**	**Ct value**	**IHC**
Brain	22.47	++	21.44	+	19.97	+++	27.54	Neg	30.48	+	20.51	+++
Cecal tonsil	23.14	++	20.55	+++	20.00	+++	31.62	Neg	29.92	Neg	22.19	+++
Heart	20.95	++	20.89	++	17.27	++++	34.34	Neg	32.29	Neg	16.49	++++
Jejunum	20.97		21.74	++	20.79	++	29.87	+	31.83	Neg	22.85	++
Kidney	21.33	+++	18.94	+++	17.25	++	27.28	+	31.20	Neg	19.87	++
Liver	16.19	++++	17.65	++++	21.27	+++	30.57	+	33.46	+	23.30	++
Lung	25.79	++++	21.25	+++	24.34	++++	25.02	+	32.07	+	23.09	++++
Muscle	23.87	+	23.60	+	21.48	+	29.65	+	28.71	+	19.56	++
Pancreas	22.75		20.54	++	23.75	++	25.65	++	35.27	Neg	28.80	+
Proventriculus	NA	++	22.46	+++	19.24	+++	31.57	Neg	31.61	Neg	23.52	+++
Spleen	23.84	++++	21.19	++++	NA	++++	34.00	+	31.47	+	21.75	++++
Trachea	24.01	+	22.66	++	20.53	++	22.83	+++	28.76	Neg	23.34	++

^1^Crossing threshold for influenza A virus matrix gene real-time RT-PCR assay.

^+^mild immunostaining (<25% of the section); ^++^moderate immunostaining (25% to 50% of the section); ^+++^abundant immunostaining (51% to 75% of the section); ^++++^intense immunostaining (>75% of the section).

Neg – negative for immunostaining; NA – not available; dpi – days post-inoculation; dpc – days post-contact.
